# Supplementary material for: New Meloxicam Derivatives—Synthesis and Interaction with Phospholipid Bilayers Measured by Differential Scanning Calorimetry and Fluorescence Spectroscopy
Source: Membranes (Basel). 2023 Apr 6;13(4):416. doi: 10.3390/membranes13040416 (PMC10145084; doi:10.3390/membranes13040416)
Supplement: Supplementary file 1 [file membranes-13-00416-s001.zip › membranes-2269222-supplementary.pdf]

Content:

1. ^{1}H NMR spectra of new compounds (pages S2-S6)

2. Computational studies:

Table S1. Absorption and distribution of studied compounds and meloxicam (MLX); page S7

Table S2. Metabolism and excretion of studied compounds and meloxicam (MLX); page S7

Table S3. Toxicity of studied compounds and meloxicam (MLX); page S8

^{1}H NMR spectra of new compounds

compound	structure	page
**PR23**	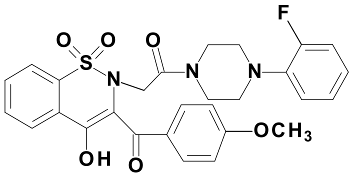	S2
**PR24**	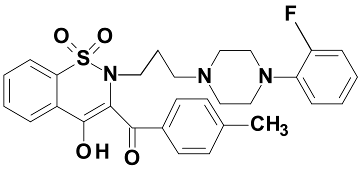	S3
**PR25**	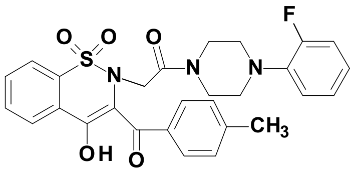	S4
**PR49**	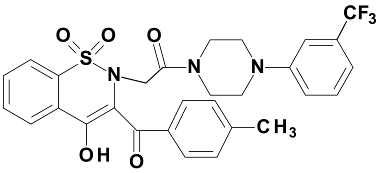	S5
**PR50**	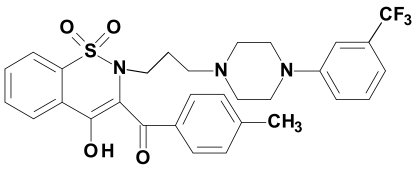	S6

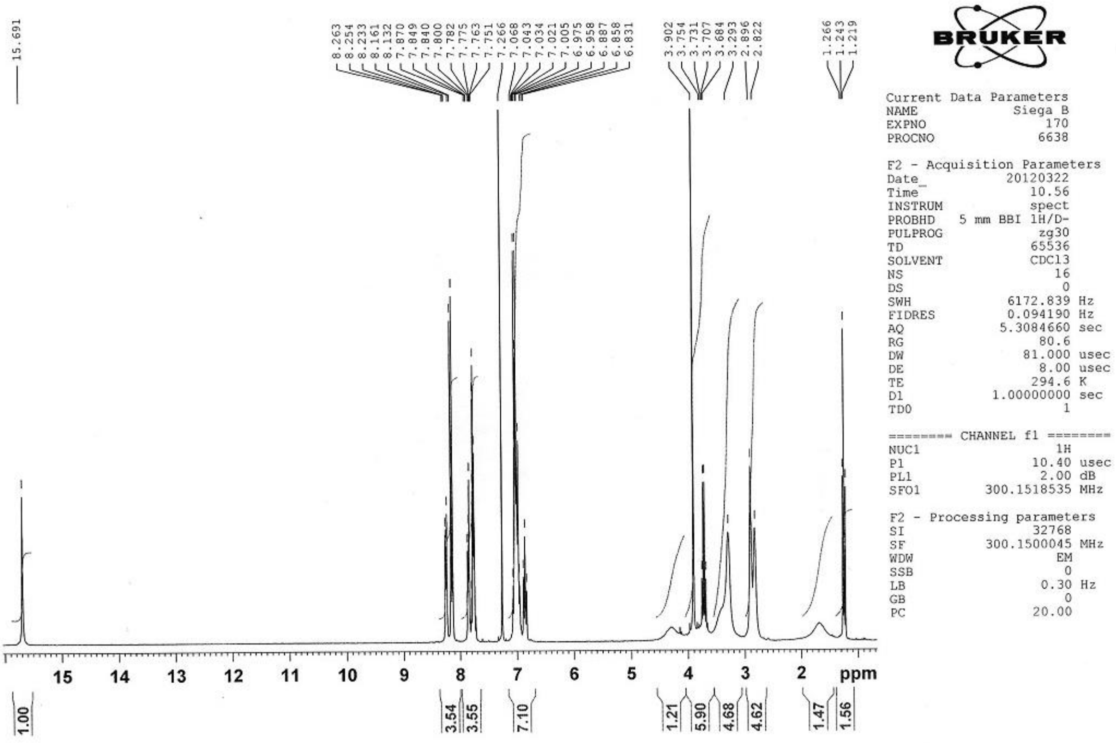

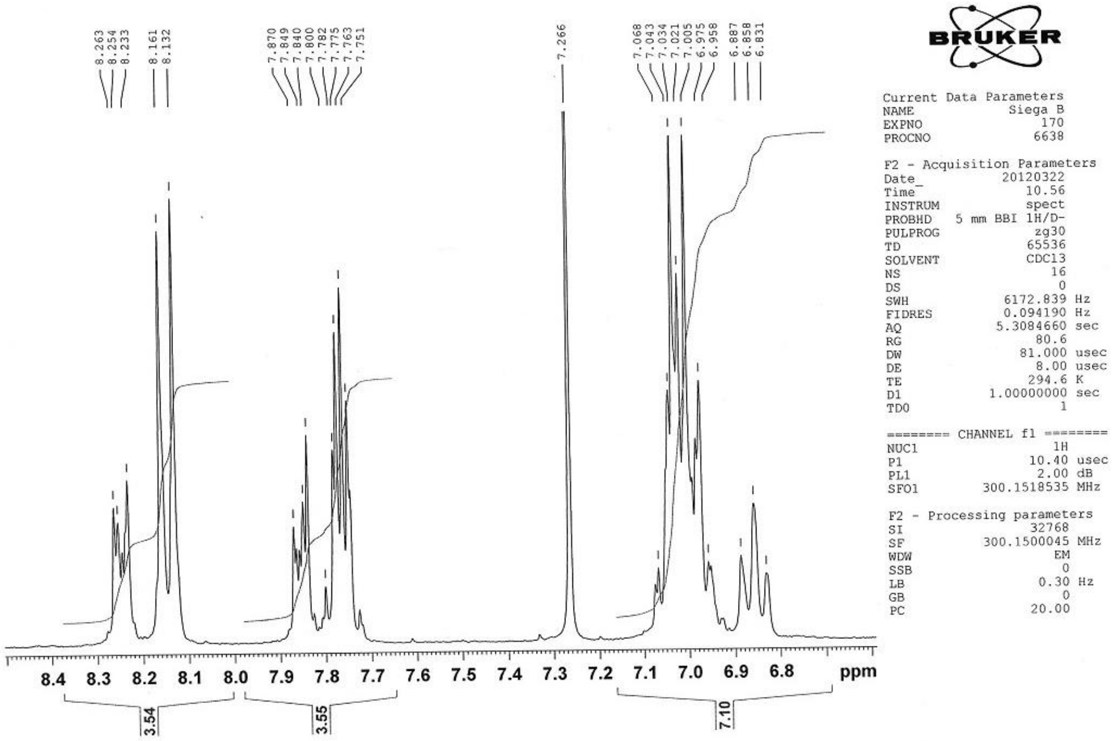

Figure S1. ^{1}H NMR spectra of **PR23** (full range at the top, aromatic range at the bottom).

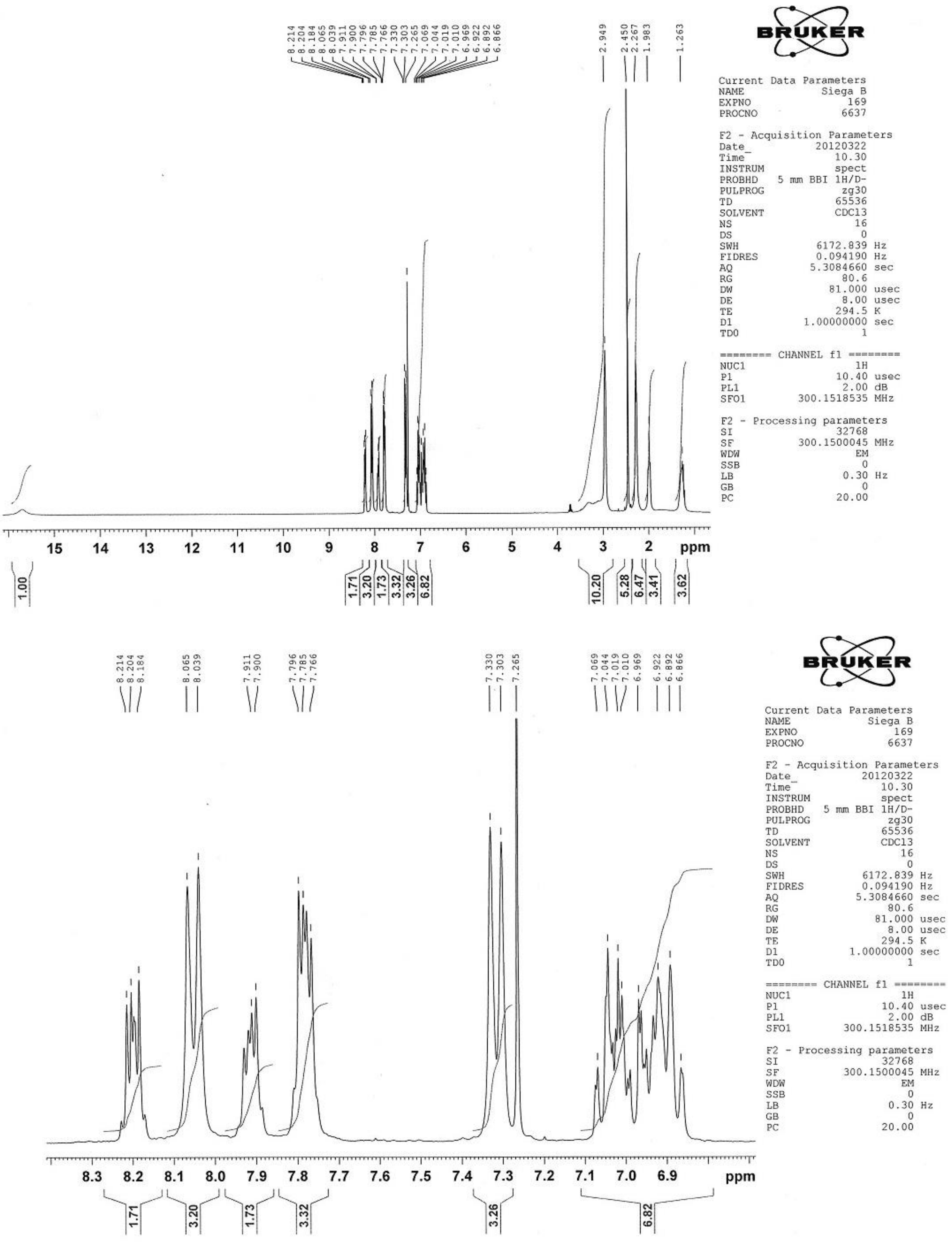

Figure S2. ^{1}H NMR spectra of **PR24** (full range at the top, aromatic range at the bottom).

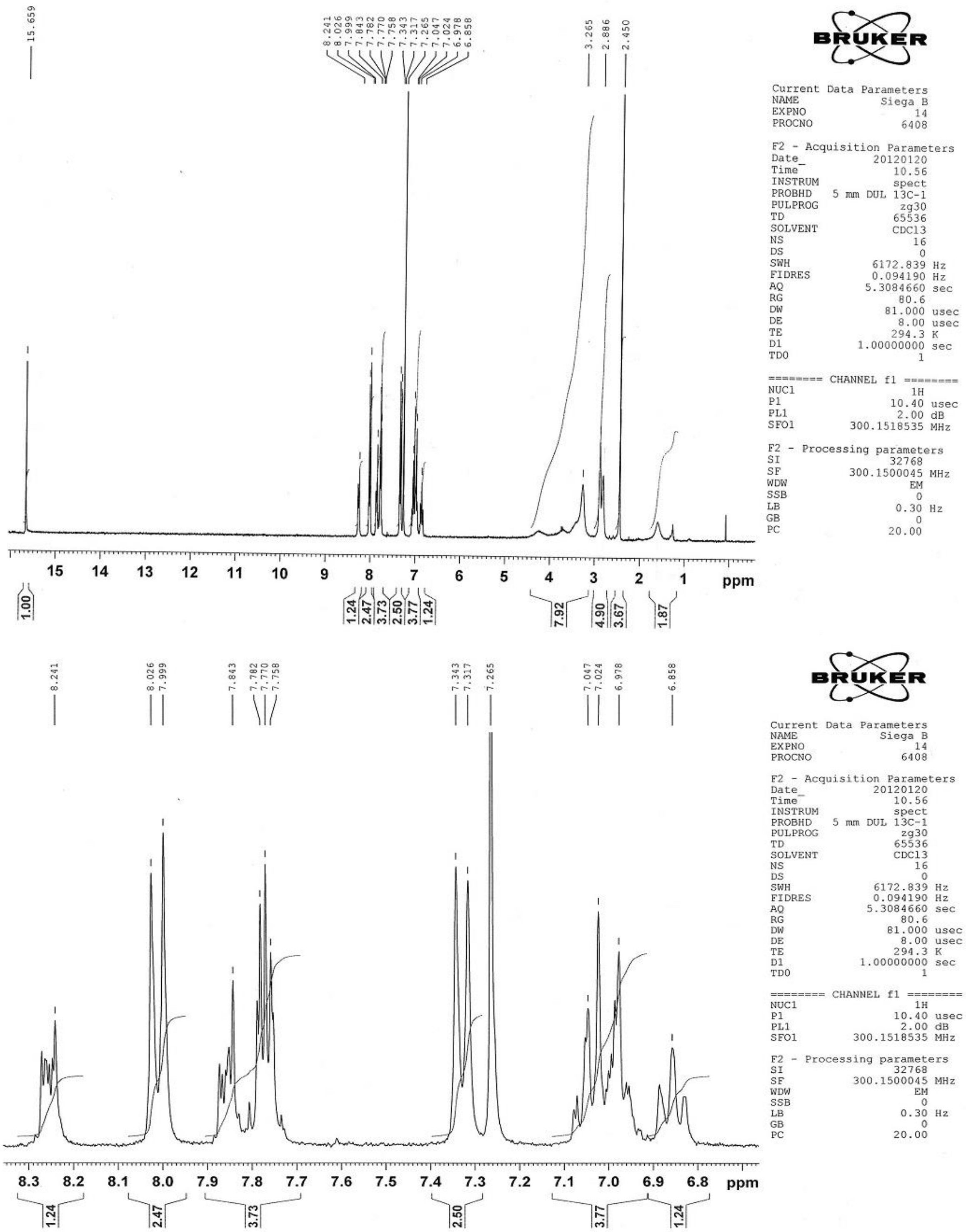

Figure S3. ^{1}H NMR spectra of **PR25** (full range at the top, aromatic range at the bottom).

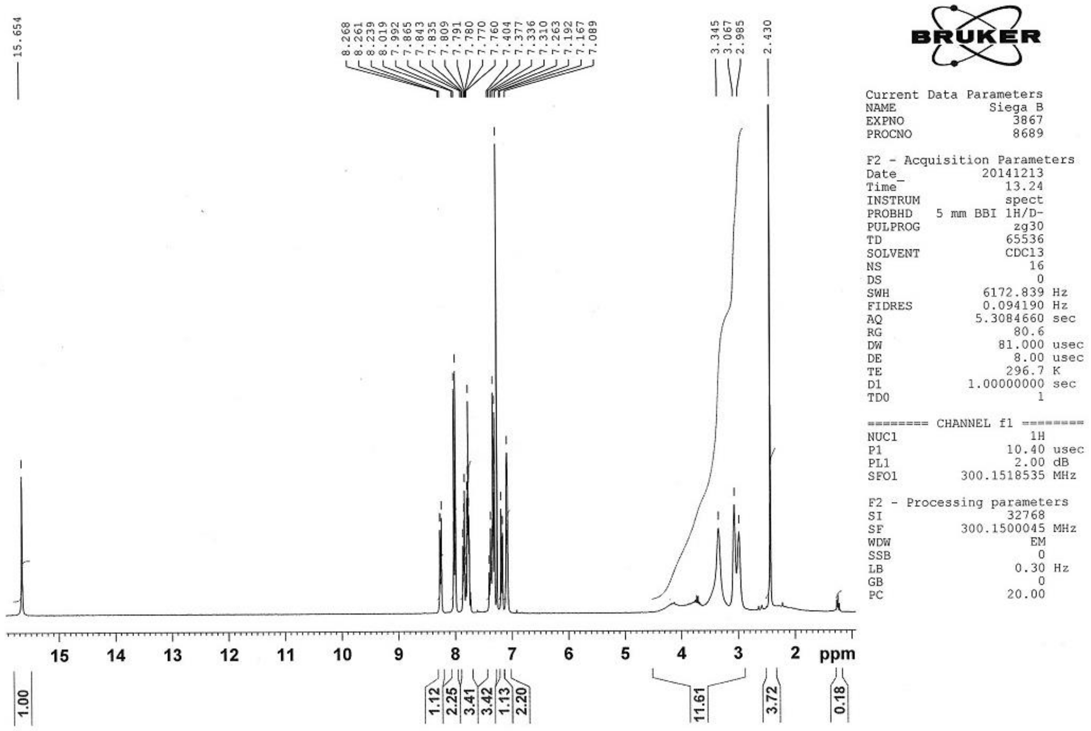

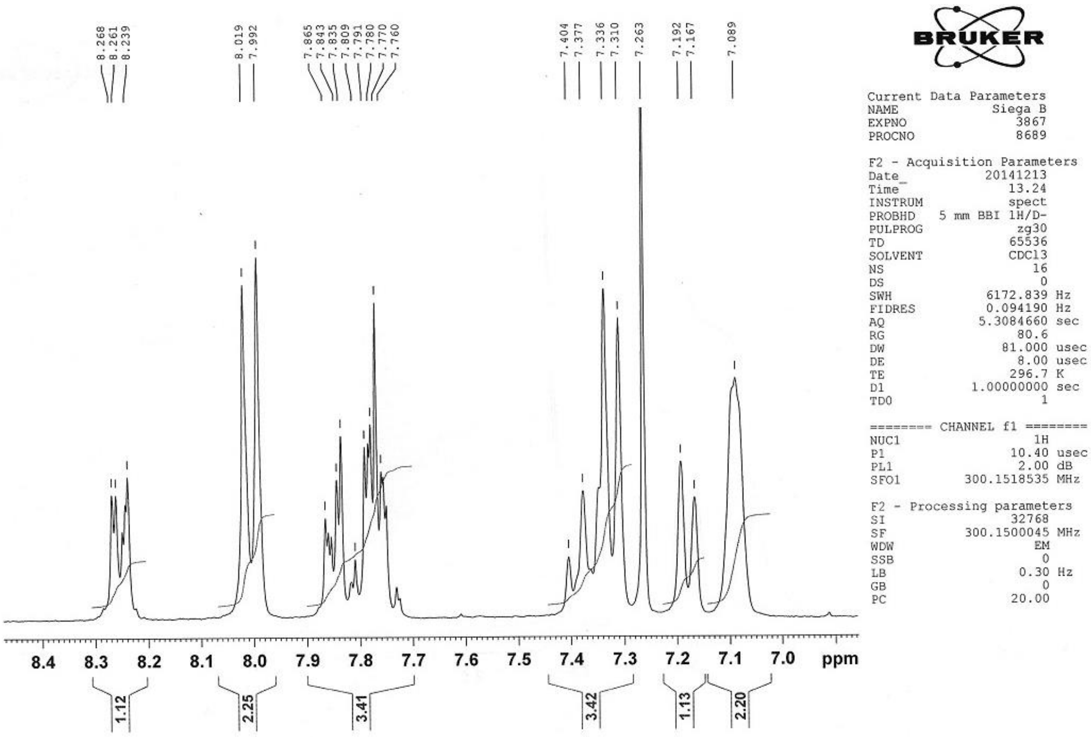

Figure S4. ^{1}H NMR spectra of **PR49** (full range at the top, aromatic range at the bottom).

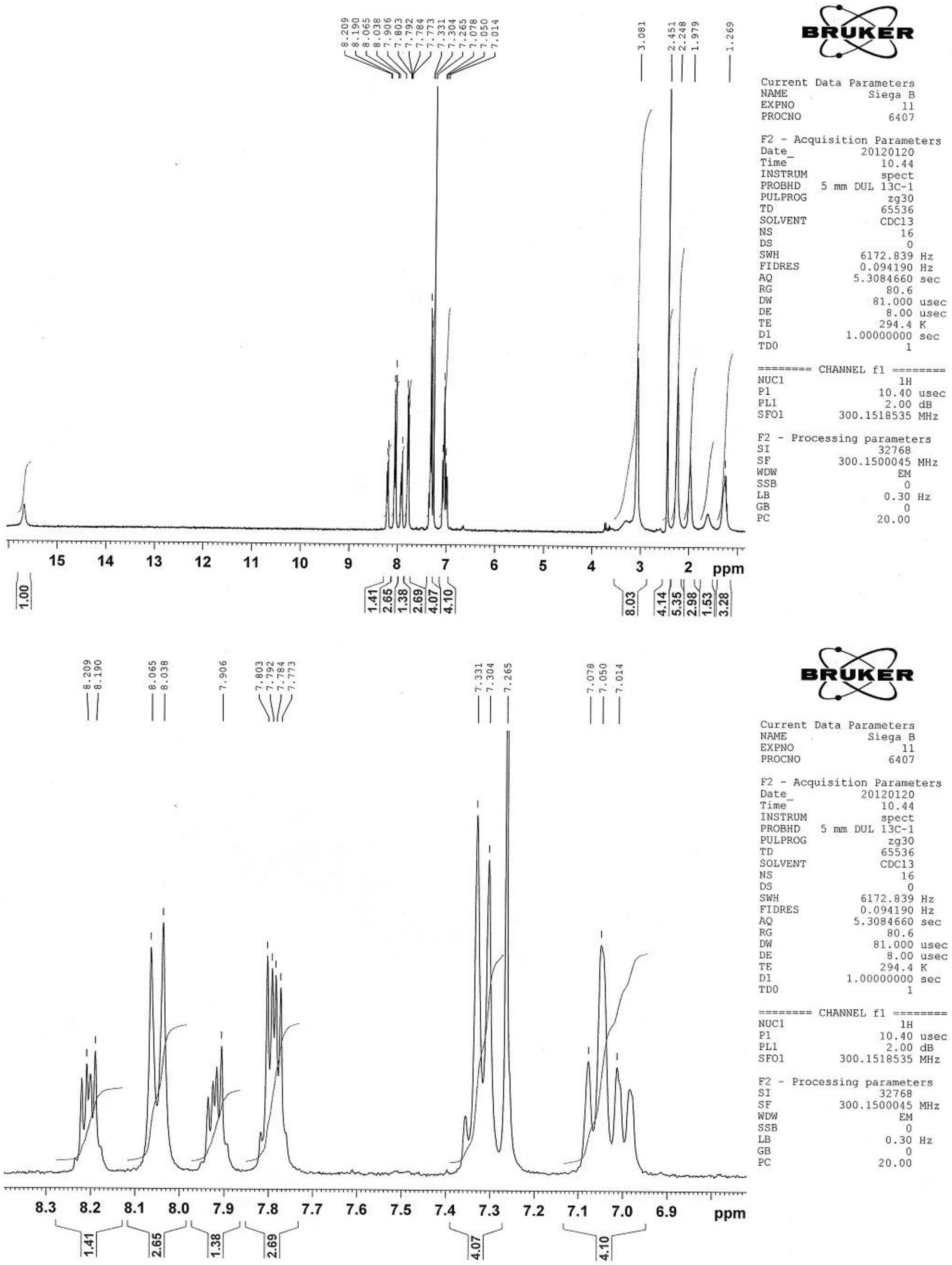

Figure S5. ^{1}H NMR spectra of **PR50** (full range at the top, aromatic range at the bottom).

Table S1. Absorption and distribution of studied compounds and meloxicam (MLX).

Parameter	Compound					
	PR23	PR24	PR25	PR49	PR50	MLX
**HIA** (Human Intestinal Absorption); Category 1: HIA+(HIA < 30%); Category 0: HIA-(HIA < 30%); The output value is the probability of being HIA+)	0.01	0.004	0.005	0.009	0.005	0.004
**Caco-2 Permeability**; optimal higher than −5.15 log cm/s	−5.5	−5.6	−5.6	−5.584	−5.7	−4.71
**MDCK Permeability**; high passive permeability: $>20\cdot10^{-6}$	$3.2\cdot10^{-5}$	$2.9\cdot10^{-5}$	$2.7\cdot10^{-5}$	$2.3\cdot10^{-5}$	$1.9\cdot10^{-5}$	$1.8\cdot10^{-5}$
**Pgp-inhibitor**	+	+	+	+	+	-
**Pgp-substrate**	-	-	-	-	-	-
**PPB** (Plasma Protein Binding; optimal < 90%. Drugs with high protein-bound may have a low therapeutic index)	98%	98%	99%	99%	98%	99%
**VD** (Volume Distribution; optimal 0.04–20 L/kg)	0.4	1.7	0.4	0.5	2.0	0.4
**BBB Penetration** (Blood-Brain Barrier Penetration Category; 1: BBB+; Category 0: BBB−; The output value is the probability of being BBB+)	0.1	0.4	0.1	0.1	0.5	0.1

Table S2. Metabolism and excretion of studied compounds and meloxicam (MLX).

Parameter	Compound					
	PR23	PR24	PR25	PR49	PR50	MLX
**CYP1A2** inhibitor	no	no	no	no	no	no
**CYP2C19** inhibitor	yes	yes	yes	yes	yes	no
**CYP2C9** inhibitor	yes	yes	yes	yes	yes	yes
**CYP2D6** inhibitor	no	yes	no	no	yes	no
**CYP3A4** inhibitor	yes	yes	yes	yes	yes	no
**CL—Clearance** (high: > 15 mL/min/kg; moderate: 5–15 mL/min/kg; low < 5 mL/min/kg)	5.3	5.1	3.8	2.9	4.2	3.867
**T ½** (category 1: long half-life > 3h; category 0: short half-life < 3h)	0.08	0.01	0.06	0.05	0.01	0.427

Table S3. Toxicity of studied compounds and meloxicam (MLX).

Parameter	Compound					
	PR23	PR24	PR25	PR49	PR50	MLX
**hERG Blockers** (heart rhythm disturbances) 1: active; 0: inactive	0.2	0.8	0.1	0.2	0.8	0.9
**H-HT—Human Hepatotoxicity** 1: positive(+); 0: negative(−)	1.0	1.0	0.9	1.0	0.9	0.9
**DILI—Drug Induced Liver Injury** 1: drugs with a high risk; 0: drugs with no risk.	1.0	1.0	1.0	1.0	1.0	1.0
**AMES Toxicity** 1: Ames positive(+); 0: Ames negative(−)	0.06	0.3	0.05	0.2	0.3	0.03
**Rat Oral Acute Toxicity** 0: low toxicity; 1: high toxicity	0.6	0.2	0.1	0.8	0.4	0.3
**Skin Sensitization** 1: sensitizer; 0: non-sensitizer	0.04	0.03	0.04	0.04	0.02	0.07
**Carcinogen** 1: carcinogens; 0: non-carcinogens	0.5	0.1	0.5	0.1	0.1	0.7
**Eye Irritation** 1: irritants; 0: non-irritants	0.006	0.006	0.006	0.003	0.006	0.011
**Respiratory Toxicity** 1: toxicants; 0: non-toxicants	0.07	0.13	0.1	0.3	0.4	0.7
